# CAM/TMA-DPH as a promising alternative to SYTO9/PI for cell viability assessment in bacterial biofilms

**DOI:** 10.3389/fcimb.2024.1508016

**Published:** 2025-01-21

**Authors:** Tinatini Tchatchiashvili, Mateusz Jundzill, Mike Marquet, Kamran A. Mirza, Mathias W. Pletz, Oliwia Makarewicz, Lara Thieme

**Affiliations:** ^1^ Institute of Infectious Disease and Infection Control, Jena University Hospital, Friedrich-Schiller-University Jena, Jena, Germany; ^2^ Institute of Infectious Disease and Infection Control, Member of the Leibniz Center for Photonics in Infection Research (LPI), Jena, Germany

**Keywords:** biofilm, viability, staining, metabolism, calcein acetoxymethyl, live/dead assay

## Abstract

**Introduction:**

Accurately assessing biofilm viability is essential for evaluating both biofilm formation and the efficacy of antibacterial treatments. Traditional SYTO9 and propidium iodide (PI) live/dead staining in biofilm viability assays often ace challenges due to non-specific staining, limiting precise differentiation between live and dead cells. To address this limitation, we investigated an alternative staining method employing calcein acetoxymethyl (CAM) to detect viable cells based on esterase activity, and 1-(4-trimethylammoniumphenyl)-6-phenyl-1,3,5-hexatriene p-toluenesulfonate (TMA-DPH) to assess the remaining biofilm population.

**Methods:**

Biofilms of *Pseudomonas aeruginosa, Klebsiella pneumoniae*, *Staphylococcus aureus*, and *Enterococcus faecium* were matured and exposed to varying concentrations of antibiotics or sterile medium. Biofilm viability was assessed using CAM/TMA-DPH or SYTO9/PIstaining, followed by analysis with confocal laser scanning microscopy (CLSM) and ImageJ-based biofilm surface coverage quantification. Viability findings were compared with colony-forming units (CFU/mL), a standard microbial viability measure.

**Results:**

CAM/TMA-DPH staining demonstrated strong positive correlations with CFU counts across all bacterial species (*r *= 0.59 - 0.91), accurately reflecting biofilm vitality. In contrast, SYTO9/PI staining consistently underestimated the viability of untreated biofilms, particularly in *Klebsiella pneumoniae*, where a negative correlation with CFU/mL was observed (*r *= –0.04). Positive correlations for SYTO9/PI staining were noted in other species (*r *= 0.65 - 0.79). These findings underscore the limitations of membrane integrity-based staining methods and highlight the advantages of metabolic-based probes like CAM/TMA-DPH.

**Discussion:**

Our findings suggest that CAM/TMA-DPH staining provides a promising alternative to SYTO9/PI for cell viability assessment in bacterial biofilms, highlighting the advantages of metabolic-based probes over traditional membrane integrity assays. The consistency of CAM/TMA-DPH staining across different bacterial species underscores its potential to advance studies on biofilm and contribute to the development of more effective anti-biofilm treatments, which is essential for clinical management of biofilm-associated infections.

## 1 Introduction

Biofilms represent complex microbial aggregates enclosed within a self-produced extracellular polymeric substance (EPS) (Donlan, 2002), providing structural stability and protection to the bacterial communities in various environments (Kim and Lee, 2016). Biofilms are associated with numerous tissue and device-related infections, such as native and prosthetic valve endocarditis or cystic fibrosis (CF) (Costerton et al., 1999). Bacteria in biofilms demonstrate increased tolerance to antibiotics and the immune system, compared to their planktonic (free-living) counterparts (Fux et al., 2005). This heightened phenotypic resistance is partly due to the EPS, which acts as a barrier that impedes antibiotic penetration and restricts immune cell access. Moreover, altered gene expression and metabolic changes within the biofilm contribute to heterogeneous cell populations with diverse physiological states (Rani et al., 2007), a key factor in their resilience and treatment resistance (Sadiq et al., 2017). Addressing biofilm-associated infections therefore remains a significant challenge, leading to ongoing research to develop compounds that can effectively disrupt and eliminate biofilms (Taylor et al., 2014; Li and Lee, 2017). Accurate *in vitro* assessment of bacterial cell viability within biofilms is essential for determining the efficacy of these anti-biofilm agents during drug development (Costerton et al., 1999).

Biofilm research employs a variety of microbiological, chemical, physical, and molecular techniques, each with distinct advantages and limitations (Azeredo et al., 2017). Among the most utilized quantitative biochemical methods are crystal violet (CV) staining, or colorimetric metabolic assays based on tetrazolium salts. CV targets the biomass of biofilms, specifically binding to negatively charged molecules, such as acidic polysaccharides and other components in the biofilm matrix without distinguishing between viable and non-viable cells. CV staining is straightforward and widely used for quantifying biofilm biomass, but it can be error-prone due to inconsistencies in staining intensity, incomplete removal of excess dye, and variability in washing and drying steps, all of which can lead to inaccuracies in biofilm quantification (Wilson et al., 2017). Tetrazolium-based assays, such as reduction of XTT (2,3-bis(2-methoxy-4-nitro-5-sulfophenyl)-5-[(phenylamino)carbonyl]-2H-tetrazolium hydroxide) and TTC (2,3,5-triphenyl-2H-tetrazolium chloride) are widely used to assess the respiratory activity of cells within biofilms. These techniques provide efficient, indirect biofilm quantification but are limited in their ability to visualize the detailed structure and organization of biofilms. They generally have low sensitivity thresholds, which can reduce accuracy, especially for biofilms with sparse biomass or those containing anaerobic organisms (Stewart and Franklin, 2008; Azeredo et al., 2017). These limitations make these methods less effective for comprehensive biofilm analysis, particularly in studies requiring precise structural or viability information.

**Figure 1:**
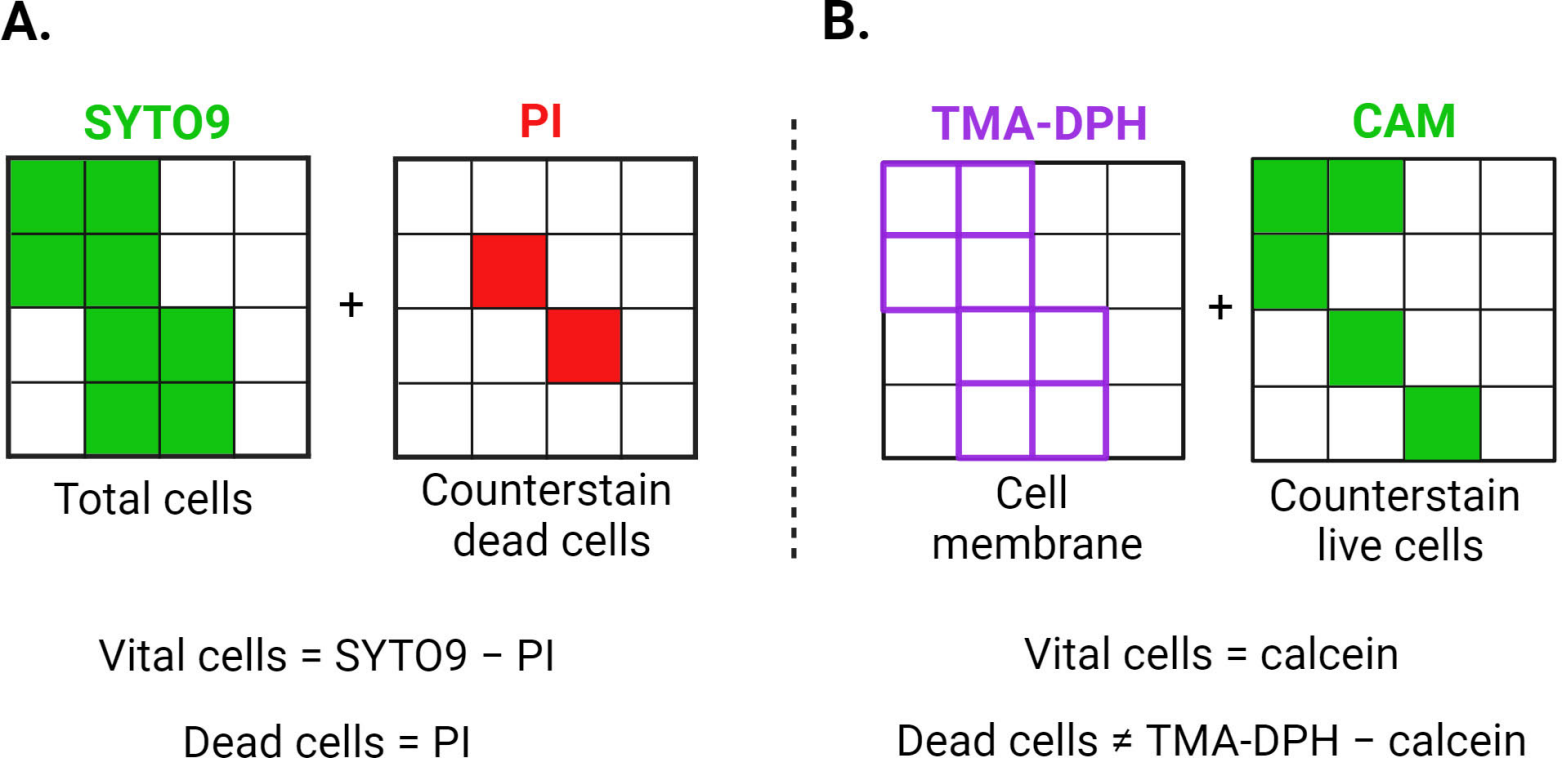
Graphical representation of the theoretical staining principles of SYTO9/PI **(A)** and CAM/TMA-DPH **(B)** dyes. One small square corresponds to one bacterium, while the entire large square represents the total image area recorded via CLSM. PI can only penetrate cells with compromised membranes, whereas SYTO9 can enter both intact and damaged cells. Both SYTO9 and PI intercalate with DNA, staining the entire cell. TMA-DPH intercalates with the lipid bilayer of bacterial membranes, staining the cell boundary without distinguishing between live and dead cells. Cellular esterases in viable cells convert CAM into fluorescent calcein, causing the entire cell to fluoresce. CAM, calcein acetoxymethyl; PI, propidium iodide; TMA-DPH, 1-(4-trimethylammoniumphenyl)-6-phenyl-1,3,5-hexatriene p-toluenesulfonate. Created in BioRender. Röll, D. (2024) BioRender.com/f07q170.

In contrast, confocal laser scanning microscopy (CLSM) provides a robust approach for biofilm analysis by enabling direct spatial visualization of biofilm architecture and its bacterial constituents. Through the use of targeted fluorescent probes, CLSM allows for specific labelling of distinct bacterial components, facilitating detailed examination of biofilm structure, organization, and cell viability (Bridier and Briandet, 2014). Commonly used fluorescent probes for evaluating biofilm vitality include SYTO9 and propidium iodide (PI), which are both nucleic acid-binding dyes. SYTO9 (green-fluorescent stain) is a membrane-permeable dye, while PI (red-fluorescent stain) penetrates only bacteria with compromised cell membranes, such as dead or dying cells, and is excluded from intact cells (Bunthof et al., 2001). In the analysis of SYTO9/PI-stained bacteria, cells emitting green fluorescence are classified as the total cell population (alive and dead), whereas those exhibiting additional red fluorescence are identified as dead. To distinguish fluorescent signals of vital cells the green and red channels should be subtracted (Boulos et al., 1999) (**Figure 1A**). However, researchers have reported several limitations of SYTO9/PI dual staining, despite its use in biofilm research for over 20 years. Both false positive and negative live/dead staining patterns have been observed in various bacterial species due to off-label target binding (Stiefel et al., 2015; Rosenberg et al., 2019), changes in membrane potential (Kirchhoff and Cypionka, 2017) or dye excretion (Minero et al., 2024). Additionally, the SYTO9/PI staining combination evaluates cell viability status by merely assessing the membrane integrity, overlooking metabolic activity. These limitations necessitate the exploration of alternative staining methods that can more accurately evaluate cell viability in biofilms.

As an alternative approach, we employed a combination of calcein acetoxymethyl (CAM) and 1-(4-trimethylammoniumphenyl)-6-phenyl-1,3,5-hexatriene p-toluenesulfonate (TMA-DPH). To the best of our knowledge, while both dyes are well-established, this is the first time these dyes have been used together for biofilm analysis. CAM, a non-fluorescent ester, permeates cell membranes and is converted by cellular esterases into fluorescent calcein, which is retained within vital cells due to its inability to cross intact membranes (Wiederschain, 2011; Tawakoli et al., 2013). This transformation enables CAM to differentiate between functionally active and inactive cells, as dead cells cannot hydrolyse or retain calcein.

To complement the visualization of remaining biofilm population, we repurposed the lipophilic fluorescent dye TMA-DPH which is commonly used in biophysics for studying cell membrane fluidity, dynamics, and lipid organization. The cationic trimethylammonium group of TMA-DPH interacts with the polar head groups of phospholipids, anchoring the dye in a specific orientation within the membrane (Yoon et al., 2011). This interaction helps TMA-DPH to stay in the membrane, making it suitable for studying membrane dynamics over time, but also for visualizing the cell membrane of both viable and non-viable bacteria (**Figure 1B**).

The study evaluated the reliability of the CAM/TMA-DPH assay in assessing biofilm viability compared to conventional SYTO9/PI staining. Mature biofilms of *Pseudomonas aeruginosa* (*P. aeruginosa*)*, Klebsiella pneumoniae* (*K*. *pneumoniae*)*, Staphylococcus aureus* (*S. aureus*), and *Enterococcus faecium* (*E. faecium*) were treated with increasing concentrations of antibiotics and stained with either CAM/TMA-DPH or SYTO9/PI. CLSM images of the biofilms were quantitatively analyzed via Biofilm Viability Checker (Mountcastle et al., 2021), and results were compared to colony-forming units (CFU/mL), the microbiological standard for assessing viability.

## 2 Materials and methods

### 2.1 Bacterial strains and antibiotics

This study used different Gram-positive and Gram-negative bacterial species available as laboratory stocks. The strains included *S. aureus* ATCC 43300, with a vancomycin minimum inhibitory concentration (MIC) of 1 mg/L; an *E. faecium* endocarditis isolate, MIBI701, exhibiting a gentamicin MIC of 32 mg/L; a mucoid *P. aeruginosa* strain, MIBI685, isolated from a cystic fibrosis patient with a ciprofloxacin MIC of 0.125 mg/L; and a bloodstream isolate of *K. pneumoniae*, IIMK 217, with a ciprofloxacin MIC of 0.0625 mg/L. The clinical isolates with various clinical backgrounds were previously collected in studies approved by the ethical committee of Jena University Hospital (3852/07-13 and 3694-02/13). MICs of vancomycin (Sigma Aldrich, St. Louis, United States), gentamicin (TCI Europe, Zwijndrecht, Belgium), and ciprofloxacin (AppliChem GmbH, Darmstadt, Germany) were determined by the broth microdilution method according to EUCAST guideline (ISO 20776-2:2021).

### 2.2 Testing of the anti-biofilm activity of selected antibiotics by CFU counting

Bacterial overnight cultures were prepared in either Todd Hewitt (TH) (*E. faecium*) or Mueller-Hinton (MH) broth (all other strains). Subsequently, the optical density (OD) was measured at 600 nm using the Thermo Scientific™ Multiskan™ GO (Thermo Fischer Scientific, Waltham, MA, USA) and adjusted to an OD of 0.08, corresponding to approximately 10^7^–10^8^ CFU/mL (Zhang et al., 2015). Biofilms were grown in triplicate by adding 200 µL of the adjusted cultures per well in 96-well flat-bottom glass microtiter plates (Greiner Bio-one, Kremsmünster, Austria). The plates were placed in a humidified chamber and incubated at 37°C with 5% CO_2_ without shaking for 48 hours. Before antibiotic treatment, the supernatants containing planktonic bacteria were carefully removed. The antibiotics were prepared as 10 mg/mL stock solutions in distilled water and freshly diluted to the needed concentrations in the respective broth (see above). As biofilms are more tolerant to antibiotics, the concentrations for biofilm treatments were chosen higher than the determined MICs. Antibiotic or mock solutions (medium only) of 200 μL per well were added for 24 h to the mature biofilms. For CFU determination, the drop plate method was employed as described by Thieme et al (Thieme et al., 2020). In brief, biofilms were washed twice with 200 µL of sterile saline to remove non-adherent cells, carefully scraped off using a loop, and serially diluted. For *S. aureus*, *E. faecium*, and *K. pneumoniae*, 10 µL aliquots of the serial dilutions were deposited in triplicate on TH or MH agar plates. In contrast, *P. aeruginosa* suspensions were conventionally plated (100 µL per plate) due to the mucoid phenotype, which results in the formation of large colonies. After overnight incubation at 37°C, bacterial colonies were counted, and CFU/mL values were calculated. Three independent biological experiments were conducted, each comprising three replicates.

### 2.3 Fluorescent staining and imaging

For fluorescent image analysis, biofilms were grown and treated as described above in parallel to the CFU assays. For fluorescent staining, an aqueous working solution of CAM and TMA-DPH (both purchased from Thermo Fisher Scientific Inc., Waltham, MA, USA) was prepared by adding 2 μL of TMA-DPH and 100 μL of CAM to 900 μL of distilled water, resulting in concentrations of 20 μM and 100 μM for TMA-DPH and CAM, respectively (Calcein AM, cell-permeant green and blue dyes, n.d). The SYTO9/PI staining working solution (FilmTracer™ LIVE/DEAD^®^ Biofilm Viability Kit, Thermo Fisher Scientific Inc.) was prepared according to the manufacturer’s protocol, with 3 μL of SYTO9 stain and 3 μL of PI stain in 1 mL of distilled water (Filmtracer™ LIVE/DEAD™ Biofilm Viability Kit, n.d). Intact, washed biofilms were subsequently stained by adding 50 μL of the respective staining solution per well. Samples were incubated for 20 min (stained with SYTO9/PI) or 60 min (stained with CAM/TMA-DPH) at room temperature, protected from light. Subsequently, the staining solutions were carefully removed, and the respective fresh broth was added to prevent the biofilms from drying up during microscopy. Toxic effects of the single dyes on the biofilms independent from the antibiotic treatment were excluded by prior CFU analysis of stained versus unstained biofilms.

Biofilms were visualized using CLSM on an LSM 980 microscope system (Carl Zeiss AG, Oberkochen, Germany), equipped with air objectives of 63×/0.65, 40×/0.65, and 20×/0.65 magnifications. Three independent microscopy experiments were conducted, each comprising three replicates. The SYTO9, CAM, and PI fluorophores were excited using an argon laser with a wavelength of 488 nm. SYTO9 and CAM emitted in the green channel at 522 nm, while PI emitted in the red channel at 635 nm. TMA-DPH was excited at a wavelength of 405 nm, with emission detected in the blue channel at 430 nm. For each sample, three observation fields were randomly selected across three replicates to ensure representativeness. An area of approximately 200 μm (X) × 200 μm (Y) was scanned at 1 μm intervals along the Z-axis (Z-stack). Z-stacks were acquired starting from the first plane where bacteria were detected. Typically, a single Z-stack was recorded per well. However, in wells where biofilms appeared non-homogeneous or exhibited variations across the well, multiple Z-stacks were captured to ensure accurate representation. The pinhole was adjusted to 0.95 Airy Units (corresponding to 1 µm). Images were processed using the ZEN 9.0 Black software package (Carl Zeiss AG).

### 2.4 Computed image analysis

The open-source tool Biofilm Viability Checker (Mountcastle et al., 2021) was used for quantitative analysis of biofilm micrographs. We adapted the original code (Mountcastle et al., 2021) to process images stained with the CAM/TMA-DPH assay (see the section 2.6). First, we adjusted the analysis to utilize the appropriate channels for CAM (green) and TMA-DPH (blue). Second, channel subtraction was excluded, as the two dyes target distinct cellular components, rendering subtraction unnecessary. Additionally, we omitted the background subtraction step, as neither dye stains extracellular DNA (eDNA). Furthermore, the tool was reprogrammed to report the absolute count of stained pixels rather than percentage values for improved accuracy. Biofilm surface area coverage was then calculated as a percentage using the formula: Surface area coverage (%) = (Biofilm covered area/Total area) × 100 (Halsted et al., 2022). The total pixel area was set at 2024 × 2024, based on the micrograph dimensions. This method was consistently applied to each image within a confocal Z-stack.

### 2.5 Statistical analysis

To assess data normality, a Shapiro-Wilk test was conducted using GraphPad Software (version 9.4.0, Boston, MA, USA), indicating a deviation from normality. All statistical analyses were then performed using the R Statistical Software (v. 4.4.1, R Core Team 2021, Vienna, Austria) using dplyr package (v. 1.1.4) (Wickham, 2014). Spearman’s rank correlation was calculated with the cor.test function (method = “spearman”) to evaluate the association between image analysis measurements and corresponding CFU values. Data visualization was carried out in GraphPad Software, and a *p-*value < 0.05 was considered statistically significant for all analyses.

### 2.6 Code availability

The macro script was adapted from (Mountcastle et al., 2021), and the modified version is available at: https://github.com/Tiktcha/Biofilm-Viability-Checker.

## 3 Results

### 3.1 Qualitative analysis of CLSM micrographs

**Figure 2:**
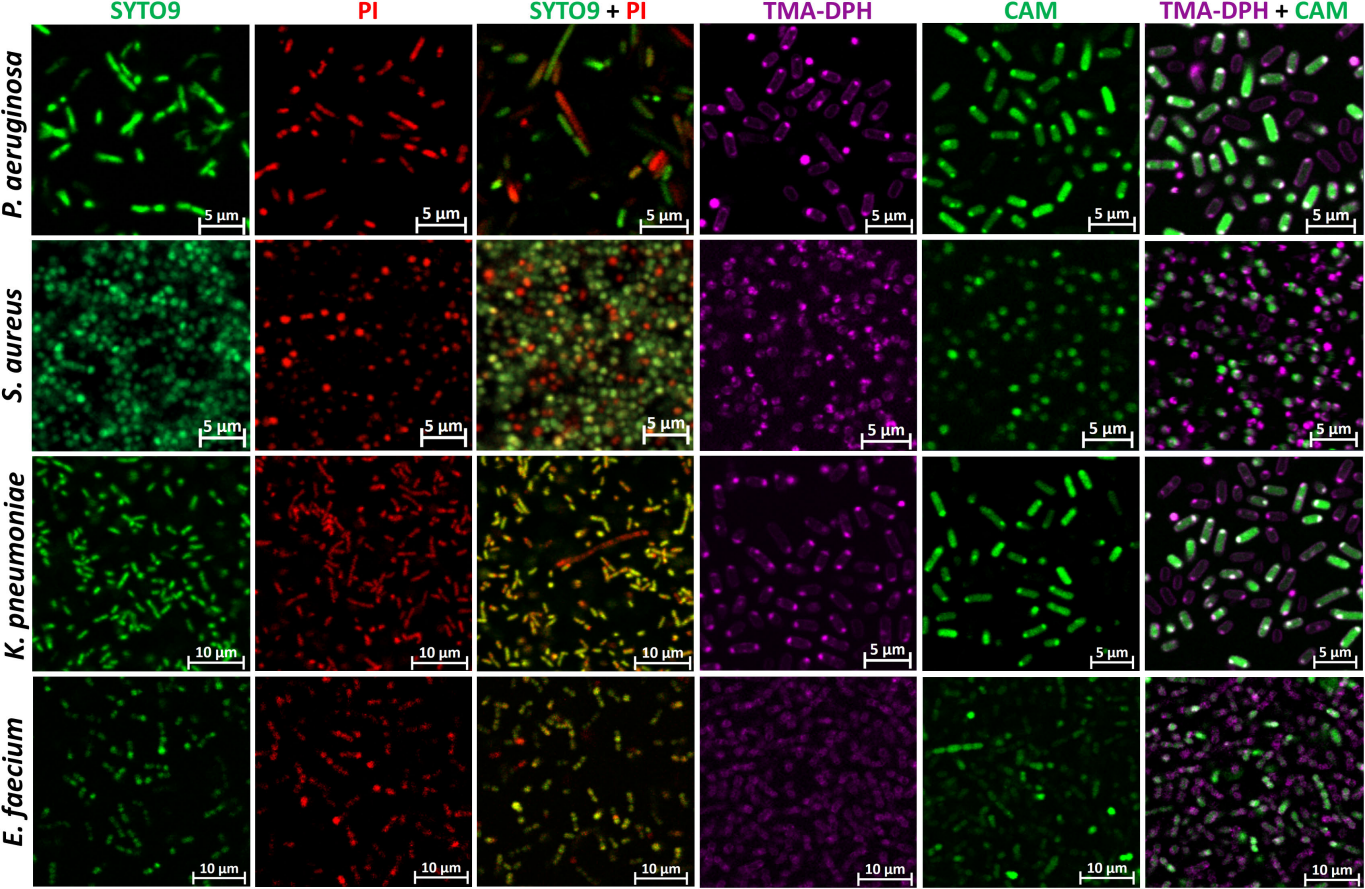
Representative CLSM images of single and combination staining of different dyes in *P. aeruginosa*, *S. aureus*, *K. pneumoniae*, and *E. faecium* biofilms. CAM, calcein acetoxymethyl; PI, propidium iodide; TMA-DPH, 1-(4-trimethylammoniumphenyl)-6-phenyl-1,3,5-hexatriene p-toluenesulfonate. Images were captured from three randomly selected fields per sample, with similar observations across all replicates. Imaging was conducted at 63× magnification.

To determine the staining quality at the individual level, CLSM images were captured at the highest resolution for single and combined staining of *P. aeruginosa, S. aureus*, *K. pneumoniae* and *E. faecium* biofilms (**Figure 2**).

In all species SYTO9 and PI exhibited strong intracellular staining, clearly delineating septum formation within the cells, which indicates ongoing cell division and membrane retraction between the developing daughter cells. Notably, in *P. aeruginosa* rods, green structures are visible under SYTO9 staining, potentially indicating the release of eDNA into the biofilm matrix. Interestingly, this phenomenon is much less pronounced with PI. In the combined staining, bacterial cells penetrated by both dyes appear yellow due to the overlap of the two fluorescence channels. However, distinct populations of cells are observed: those stained predominantly red (presumably dead) and those stained green (presumably alive). Notably, red fluorescence appears to be leaking from some cells visible as a red blur, which is indicative of compromised membrane integrity, a hallmark of cell death.

Using the membrane-specific dye TMA-DPH, the outer cell layer of all species could be specifically visualized as violet-colored cell borders. In contrast, the CAM dye produced an almost homogeneous intracellular staining. However, in some cells, the staining appeared uneven, suggesting possible septum formation. Interestingly, in Gram-negative species, combined staining with CAM and TMA-DPH revealed that the violet membrane dye signal was barely visible when cells exhibited strong green fluorescence from CAM, clearly highlighting them (**Figure 2**). In contrast, for Gram-positive species, the hypothesized outcome was observed during combined staining: CAM stained the cytoplasm, while TMA-DPH marked the membrane.

### 3.2 Qualitative analysis of biofilm micrographs after antibiotic treatment

**Figure 3:**
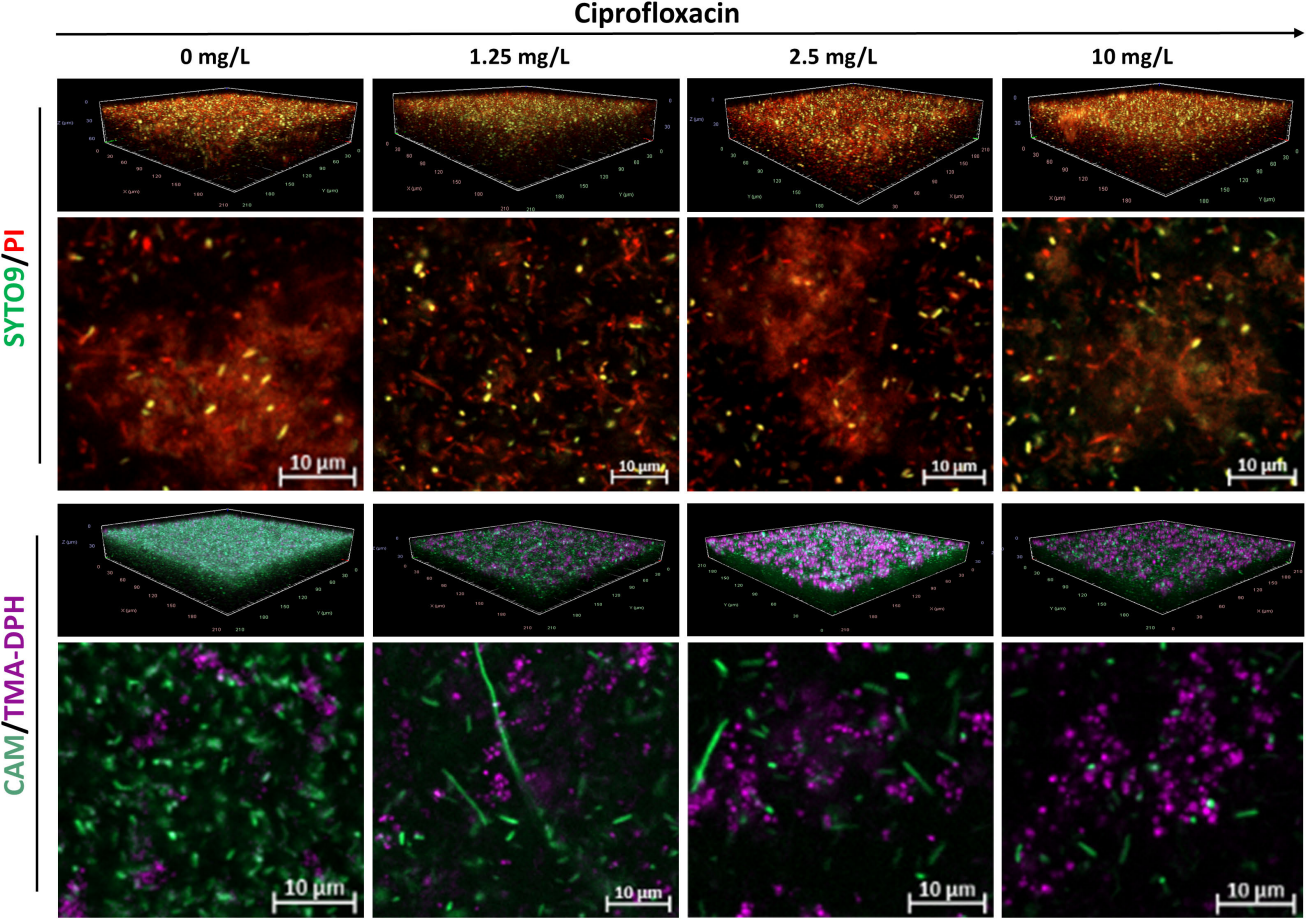
Representative CLSM images of *P. aeruginosa* MIBI685 biofilms treated with increasing concentrations of ciprofloxacin. Shown are 3D visualizations of the biofilms and representative X-Y intersections, stained with SYTO/PI (upper panel) or CAM/TMA-DPH (lower panel). CAM, calcein acetoxymethyl; PI, propidium iodide; TMA-DPH, 1-(4-trimethylammoniumphenyl)-6-phenyl-1,3,5-hexatriene p-toluenesulfonate. Images were captured from three randomly selected fields per sample, with similar observations across all replicates. Imaging was conducted at 40× magnification.

The staining efficacy of SYTO9/PI and CAM/TMA-DPH was evaluated at the population level in both untreated and antibiotic-treated biofilms. Untreated *P. aeruginosa* biofilms stained with SYTO9/PI exhibited a weak SYTO9 and strong PI signal, contrasting with strong CAM fluorescence observed in biofilms stained with CAM/TMA-DPH (**Figure 3**). As expected, increasing concentrations of ciprofloxacin led to a progressive decrease in CAM-stained cells, while concurrently increasing the fluorescence intensity of TMA-DPH. Conversely, *P. aeruginosa* biofilms stained with SYTO9/PI, showed thick aggregates of PI-stained matrix components and cells regardless the antibiotic treatment (**Figure 3**).

In the CAM and PI channels, morphological changes toward elongated rod shapes were observed in response to ciprofloxacin treatment. Both the length and the number of these cells progressively decreased with increasing concentrations of ciprofloxacin.

**Figure 4:**
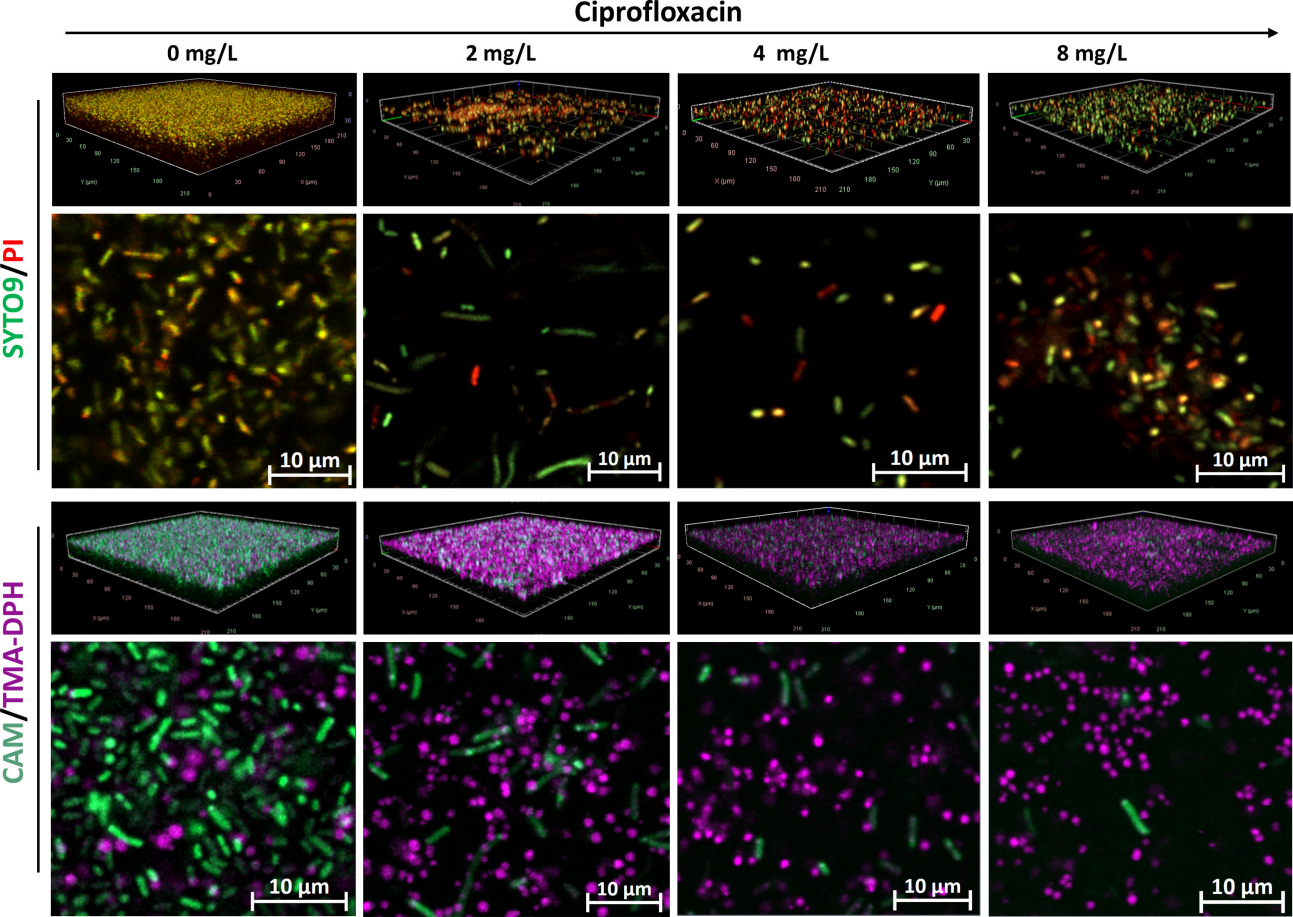
Representative CLSM images of *K. pneumoniae* IIMK217 biofilms treated with increasing concentrations of ciprofloxacin. Shown are 3D visualizations of the biofilms and representative X-Y intersections, stained with SYTO/PI (upper panel) or CAM/TMA-DPH (lower panel). CAM, calcein acetoxymethyl; PI, propidium iodide; TMA-DPH, 1-(4-trimethylammoniumphenyl)-6-phenyl-1,3,5-hexatriene p-toluenesulfonate. Images were captured from three randomly selected fields per sample, with similar observations across all replicates. Imaging was conducted at 40× magnification.

In untreated *K. pneumoniae* samples, SYTO9/PI-stained micrographs displayed a dense biofilm architecture characterized by red/yellowish fluorescence, whereas antibiotic-treated biofilms exhibited significant disruption of the biofilm structure, with no observable staining of matrix components (**Figure 4**). Conversely, *K. pneumoniae* biofilms stained with CAM/TMA-DPH showed a decrease in calcein fluorescence and an increase in TMA-DPH fluorescence with increasing concentrations of ciprofloxacin, similar to the observations in *P. aeruginosa* biofilms. Also here, morphological changes to elongated rods were observed due to ciprofloxacin treatment, but those were visible in the CAM and SYTO9 Channels (**Figure 4**).

**Figure 5:**
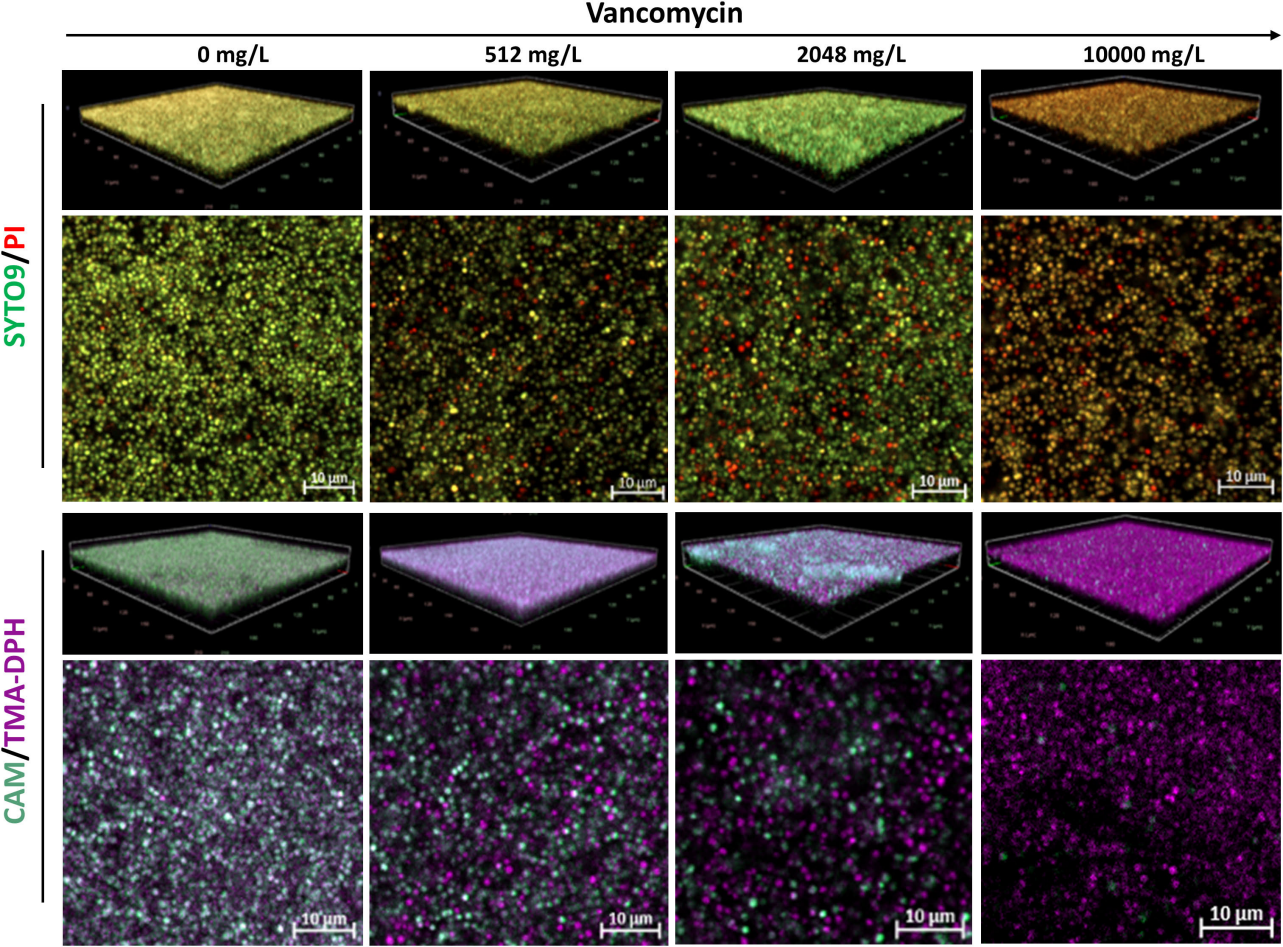
Representative CLSM images of S. aureus ATCC 43300 biofilms treated with increasing concentrations of vancomycin. Shown are 3D visualizations of the biofilms and representative X-Y intersections, stained with SYTO/PI (upper panel) or CAM/TMA-DPH (lower panel). CAM, calcein AM; PI, propidium iodide; TMA-DPH, 1-(4-trimethylammoniumphenyl)-6-phenyl-1,3,5-hexatriene p-toluenesulfonate. Images were captured from three randomly selected fields per sample, with similar observations across all replicates. Imaging was conducted at 40× magnification.

Unlike Gram-negative species, untreated *S. aureus* biofilms stained with SYTO9/PI showed strong green and weak red fluorescence (**Figure 5**). As antibiotic concentrations increased, the PI signal became more intense. Remarkably, *S. aureus* biofilms treated with 2048 mg/L of vancomycin exhibited unusually intense SYTO9 fluorescence. Furthermore, untreated biofilms stained with CAM/TMA-DPH displayed strong green fluorescence, which predictably diminished as antibiotic concentrations increased (**Figure 5**).

**Figure 6:**
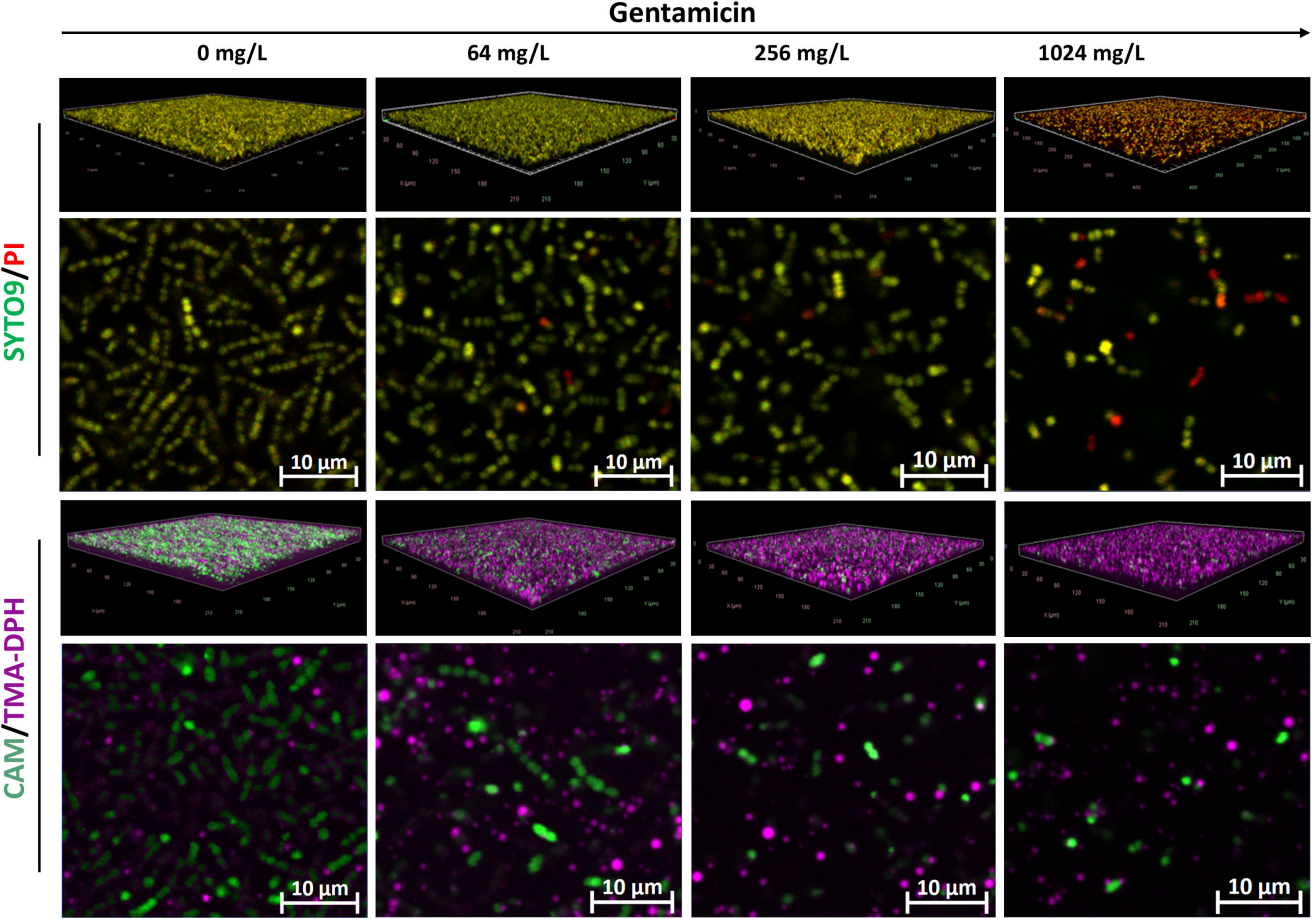
Representative CLSM images of *E. faecium* MIBI701 biofilms treated with increasing concentrations of gentamicin. Shown are 3D visualizations of the biofilms and representative X-Y intersections, stained with SYTO/PI (upper panel) or CAM/TMA-DPH (lower panel). CAM, calcein acetoxymethyl; PI, propidium iodide; TMA-DPH, 1-(4-trimethylammoniumphenyl)-6-phenyl-1,3,5-hexatriene p-toluenesulfonate. Images were captured from three randomly selected fields per sample, with similar observations across all replicates. Imaging was conducted at 40× magnification.

Compared to other species, *E. faecium* exhibited relatively weaker biofilm formation (**Figure 6**). Interestingly, similar staining patterns were observed between the SYTO9/PI and CAM/TMA-DPH staining techniques. Untreated biofilms stained with either SYTO9/PI or CAM/TMA-DPH predominantly exhibited green fluorescence but lacked a dense structure or eDNA, as indicated by PI staining. The green fluorescence progressively diminished with increasing gentamicin concentrations, correlating with the disruption of biofilm structure in treated samples (**Figure 6**).

### 3.3 Computational image analysis and comparison with CFU counts

To assess biofilm viability in CLSM micrographs of untreated and antibiotic-treated biofilms that were stained with SYTO9/PI or CAM/TMA-DPH, we quantified the respective surface coverage: either as the sum of the recorded calcein fluorescence signals, or as the difference of the SYTO9 – PI signals, subtracting the PI fluorescence from the SYTO9 fluorescence.

**Figure 7:**
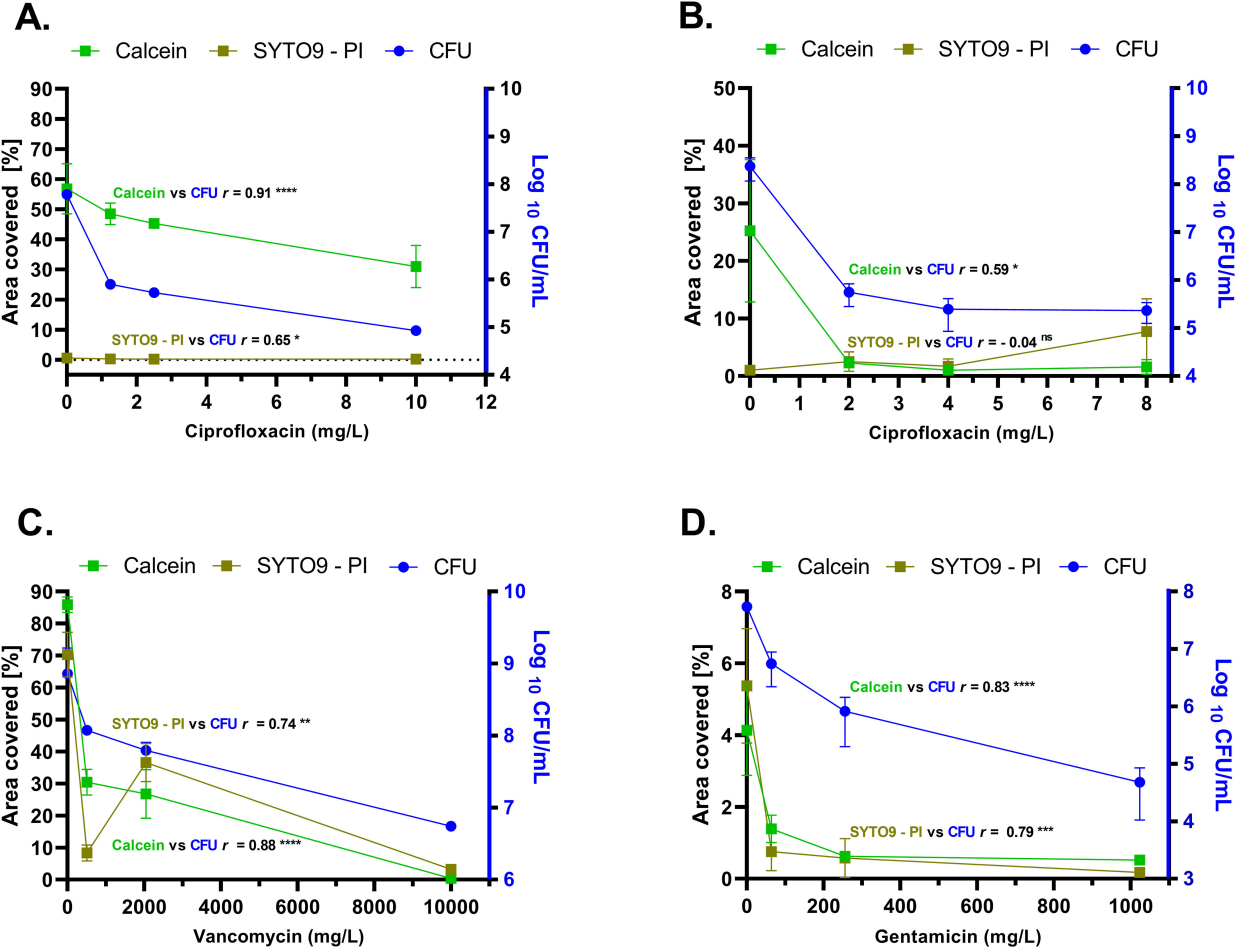
Comparative analysis of biofilm surface area coverage (left Y-axis) and number of viable cells (CFU/mL) (right Y-axis) across biofilms of *P. aeruginosa* **(A)**, *K*. *pneumoniae* **(B)**, *S. aureus* **(C)**, and *E*. *faecium* **(D)**. “SYTO9 – PI” indicates the net fluorescence differential between SYTO9 and PI signals post-subtraction. “Calcein” indicates the signal of the fluorophore calcein after digestion of CAM by intracellular esterases. The biofilm surface area coverage was calculated with the formula: Surface area coverage (%) = (Biofilm covered area/Total area) × 100. The total pixel area for analysis was equal to 2024 × 2024, derived from CLSM micrographs. Three independent experiments, each with three replicates were performed. In some cases, the error bars are too small to be visible, as they are obscured by the data symbols. CAM, calcein acetoxymethyl; CFU, colony forming unit; PI, propidium iodide; vs: versus. *r* = Spearman correlation rank. Significance was assumed at the *p* value < 0.05 and was indicated by asterisks: * < 0.05, ** < 0.01, *** < 0.001, **** < 0.0001, ns, not significant.

In untreated *P. aeruginosa* biofilms, the calcein-stained area was substantially larger than that stained by SYTO9 – PI, with respective coverage values of 56.80% ± 8.33% and 0.56% ± 0.14% (**Figure 7A**) (**Supplementary Table 1**). Similarly, at all tested ciprofloxacin concentrations, calcein-stained biofilms consistently showed higher area coverage compared to SYTO9 – PI-stained samples. A strong significant positive correlation (*r* = 0.91, *p* < 0.0001) was observed between calcein-stained area coverage and CFU counts in both untreated and treated *P. aeruginosa* biofilms. Conversely, SYTO9 – PI-stained biofilm area coverage exhibited significant but moderate correlation (*r* = 0.65, *p* = 0.02) with CFU counts (**Figure 7A**) (**Supplementary Table 1**).

Similar to *P. aeruginosa*, untreated *K. pneumoniae* biofilms showed higher calcein-stained area coverage compared to those stained with SYTO9 – PI, with mean values of 25.23% ± 12.35% and 1.0% ± 0.20%, respectively (**Figure 7B**). As the ciprofloxacin concentration increased, the calcein-stained area coverage decreased, showing a positive correlation (*r* = 0.59, *p* = 0.03) with viable cell counts obtained from CFU experiments. Contrarily, no correlation (*r* = – 0.04, *p* = 0.87) was observed between SYTO9-stained biofilm area coverage and CFU counts in *K. pneumoniae* biofilms (**Figure 7B**) (**Supplementary Table 1**). Notably, SYTO9/PI-stained biofilms treated with the highest concentration of ciprofloxacin showed greater SYTO9 – PI surface coverage compared to the untreated samples, with the mean values of 7.71% and 1.0%, respectively (**Figure 7B**) (**Supplementary Table 1**).

Similar to Gram-negative species, untreated *S. aureus* biofilms exhibited higher calcein-stained surface area coverage compared to those stained with SYTO9 – PI, albeit with a smaller difference (respective mean values of 85.85% ± 2.42% and 70.27% ± 6.99%). (**Figure 7C**). As antibiotic concentrations increased, calcein-stained surface area progressively decreased and showed a strong positive correlation (*r* = 0.88, *p* = 0.00015) with CFU counts. Interestingly, biofilms treated with 2048 mg/L vancomycin exhibited increased SYTO9 – PI area coverage, with mean value of 36.60% ± 5.94%, while biofilms stained with calcein showed lower area coverage with mean value of 26.76% ± 7.63 (**Figure 7C**) (**Supplementary Table 1**). However, there was a positive correlation between SYTO9-stained biofilm area coverage and the corresponding CFU counts (*r* = 0.74, *p* = 0.005).

In untreated *E. faecium* biofilms, areas stained with calcein or SYTO9 – PI covered approximately 4.13% ± 1.26% and 5.37% ± 1.59% of the surface area, respectively (**Figure 7D**). A strong positive correlation was found between calcein-stained area coverage and viable cell counts (*r* = 0.83, *p* = 0.00083). Similarly, a significant positive correlation was observed between SYTO9/PI-stained area and CFU counts (*r* = 0.79, *p* = 0.001) (**Figure 7D**) (**Supplementary Table 1**).

## 4 Discussion

This study aimed to assess biofilm viability across diverse bacterial species using CAM/TMA-DPH staining as an alternative to the conventional SYTO9/PI method, leveraging CAM’s ability to selectively stain live cells. The findings indicate that CAM/TMA-DPH staining demonstrates a strong correlation with viable cell counts across all tested species. This underscores the importance of incorporating assessments of cellular activity into biofilm viability evaluations, thereby addressing the limitations of membrane integrity-based methods, such as the SYTO9/PI approach.

CAM presents several advantages over SYTO9/PI staining for assessing bacterial biofilm viability. Its cleavage product, calcein, fluoresces solely within live cells due to its reliance on intracellular esterase activity (Wilson et al., 2017). This mechanism enables highly specific staining of viable cells with active metabolism. Unlike SYTO9/PI CAM’s targeted fluorescence prevents calcein from accumulating in extracellular fluid, eliminating the need for extra wash steps and improving both the accuracy and efficiency of viability measurements (Wilson et al., 2017). Notably, CAM does not bind to eDNA, thereby enhancing staining precision by exclusively targeting live cells. This contrasts with SYTO9/PI, which is prone to intercalate with eDNA in biofilms (Rosenberg et al., 2019). Furthermore, calcein provides stable fluorescence, shows strong resistant to photobleaching, and remains unaffected by pH fluctuations (Hiraoka and Kimbara, 2002). In contrast, SYTO9/PI staining is more prone to photobleaching, resulting in reduced signal intensity over time (Stiefel et al., 2015). Despite these benefits, CAM’s application in biofilm studies remains relatively uncommon.

Biofilms are highly heterogeneous communities consisting of various subpopulations with differing levels of viability (Stewart and Franklin, 2008). These subpopulations include viable but non-culturable (VBNC) cells (Pasquaroli et al., 2013; Dong et al., 2020), persisters (Lewis, 2007; Stewart and Franklin, 2008), dormant cells (Lewis, 2007), hollow membrane-enclosed vessels (Liu et al., 2023), and phenotypic variants such as small colony variants (SCVs) (Häußler, 2004). Calcein, which simultaneously targets metabolic activity and cell membrane integrity (Lichtenfels et al., 1994), can be used to detect survival subpopulations within biofilms, such as VBNC and persister cells that maintain both metabolic functions and intact membranes (Liu et al., 2023). However, a commonly employed method to study VBNC cells in biofilms is membrane integrity-based SYTO9/PI staining (Gao et al., 2021), which overlooks the fact that this method can be significantly constrained. Specifically, hollow membrane-enclosed vessels may be mistakenly identified as viable cells, reducing the accuracy of membrane-integrity-based approaches (Song and Wood, 2021) for identifying VBNC populations in biofilms.

In this study, our primary objective was to quantify the antimicrobial effects on mature biofilms, recognized as one of the most structurally complex and resilient biofilm forms, presenting substantial challenges in clinical antimicrobial testing (Römling and Balsalobre, 2012). To our knowledge, this is the first study to combine CAM and TMA-DPH staining to evaluate biofilm viability while simultaneously quantifying antimicrobial effects. We were able to visualize both the viable cells stained with CAM and the residual biofilm population co-stained with TMA-DPH to detect structural changes in cell membranes triggered by antibiotic exposure.

To address the diversity of biofilm structures, we applied a range of antibiotic concentrations, targeting biofilms at varying structural and developmental stages. The efficacy of the CAM/TMA-DPH and SYTO9/PI staining methods was assessed relative to CFU counts, which provide a more sensitive and specific measure of viability, in contrast to commonly used CV or tetrazolium salt assays. CV staining was not employed because it indiscriminately labels total biofilm biomass without differentiating between live and dead cells (Wilson et al., 2017), reducing its utility in viability-focused studies. Likewise, XTT and TTC assays were not utilized, as these reagents have demonstrated inhibitory effects on bacterial cells (Ullrich et al., 1996). Additionally, the outcomes can vary significantly due to metabolic differences among isolates and species, limiting their reliability for accurately quantifying biofilm development (Ramage, 2016).

Our results, particularly the strong correlation between CAM/TMA-DPH staining and CFU counts, is not surprising as both techniques detect active, living cells. This was particularly evident in *P. aeruginosa* and *S. aureus* biofilms, where the calcein signal exhibited a near-perfect correlation with CFU values. The decrease in calcein fluorescence with increasing antibiotic concentrations, indicating a reduction in viable cells, suggests that the CAM/TMA-DPH staining method accurately reflects the effects of antibiotics. In contrast, the SYTO9/PI staining method often failed to quantitatively capture the full extent of viability, particularly in biofilms of Gram-negative species. In *P. aeruginosa* this was likely due to high eDNA content masking viable cells (Rosenberg et al., 2019) leading to an underestimation of biofilm viability. However, the positive correlation observed in *P. aeruginosa* biofilms between SYTO9/PI staining and viable cell counts is likely attributed to the computational approach used, which helps minimize interference from EPS and non-specific staining (Mountcastle et al., 2021).

In *K. pneumoniae* biofilms, the persistence of SYTO9 fluorescence following treatment with the highest antibiotic concentration can be attributed to generally stronger binding affinity of SYTO9 to dead cells (Stiefel et al., 2015), even when counterstained with PI. The preferentially more effectively SYTO9-stained dead Gram-negative bacteria over intact ones may also result from lower membrane permeability and active efflux mechanisms of viable cells that limit dye uptake (Minero et al., 2024). This hypothesis is further supported by the lack of a significant correlation between SYTO9 – PI-stained biofilm area coverage and CFU counts. In contrast, the strong positive correlation between calcein fluorescence and culturable cell counts suggests that the CAM staining method accurately reflects the presence of viable cells in the *K. pneumoniae* biofilm.

Cell shape changes observed in Gram-negative species at lower ciprofloxacin doses suggest that the drug inhibits cell division. This effect was most pronounced at these lower concentrations, indicating that cell division inhibition occurs at doses below those required for biofilm eradication. Fluoroquinolones passively penetrate bacterial membranes (Cramariuc et al., 2012) primarily targeting DNA gyrase and topoisomerase IV, thereby inhibiting DNA replication and, consequently, cell division. At higher concentrations, however, this inhibition escalates, ultimately leading to cell death (Pommier et al., 2010) before significant morphological alterations can be established. Interestingly these phenotypes were CAM-stained in both *K. pneumoniae* and *P. aeruginosa*, but were differentially stained by SYTO9 (in *K. pneumoniae*) and PI (in *P. aeruginosa*). We cannot clearly explain this observation but due to the calcein fluorescence in those phenotypes, we must assume activity and viability.

In antibiotic-treated *S. aureus* biofilms, the intriguing shift with increased SYTO9 – PI-stained area coverage at 2048 mg/L vancomycin could be attributed to *S. aureus’* ability to enter a dormant state after antibiotic exposure (Pasquaroli et al., 2013), during which cells maintain intact membranes but exhibit reduced metabolic activity (Li et al., 2014). Since SYTO9 uptake is independent of the membrane potential, it can stain dormant cells, keeping the SYTO9 signal relatively high despite the reduced activity. This can explain the decreased fluorescence of calcein while the SYTO9 signal remained relatively high.

In this study, the weak biofilm formation and limited EPS production observed in *E. faecium* likely explain the comparable performance of both vital staining techniques. As a result, both the SYTO9/PI and CAM/TMA-DPH methods were found to be effective for assessing biofilm viability in *E. faecium*.

The enhanced detection of simultaneous fluorescence from CAM and TMA-DPH co-staining in Gram-positive bacteria, compared to Gram-negative species, can be attributed to structural and functional differences between Gram-positive and Gram-negative bacteria, particularly in their cell wall composition and efflux mechanisms. It was demonstrated that multiple xenobiotic efflux pumps, in combination with the outer membrane permeability barrier, play a critical role in *P. aeruginosa* by expelling fluorescent probes (Germ et al., 1999). Similarly, Ocaktan et al. emphasized the synergy between the efflux pump and outer membrane permeability in reducing the intracellular concentrations of TMA-DPH in *P. aeruginosa* (Ocaktan et al., 1997). These findings support our hypothesis that the presence of active metabolic processes in *P. aeruginosa* may trigger an efflux mechanism that prevents effective TMA-DPH accumulation in calcein-stained cells. This interaction may be absent or minimal in Gram-positive *S. aureus*, which lacks an outer membrane and has a thicker peptidoglycan layer that is more permeable to cationic molecules (Lambert, 2002). As a result, both TMA-DPH and CAM can penetrate and accumulate within *S. aureus* cells, leading to detectable fluorescence signals. The differences in the staining effectiveness of TMA-DPH and CAM in Gram-positive and Gran-negative species could potentially lead to misinterpretations. However, the comparison with CFU counts indicates that this bias is negligible, at least under the conditions of our experimental setup.

Previous studies comparing fluorescence-based assays with CFU measurements for quantifying adherent bacteria (Hannig et al., 2007; Tawakoli et al., 2013) were unable to establish a correlation between the two methods, likely due to computational approaches used for viability assessment. In contrast, our study utilized an ImageJ-based automated image analysis method, the Biofilm Viability Checker (Mountcastle et al., 2021), which quantifies biofilm cell vitality by analyzing stained pixels in CLSM micrographs. The software enables image preprocessing and automated thresholding, specifically accounting for the presence of eDNA by removing uniform, consistent backgrounds from images, distinguishing it from other biofilm analysis tools (Mountcastle et al., 2021). Although the original code was developed for SYTO9/PI-stained confocal micrographs, we adapted it to support CAM/TMA-DPH staining. (Detailed modifications are provided in Section 2.4, and the link of updated macro code is available in Section 2.6).

Accurately assessing biofilm viability is crucial for evaluating the efficacy of antibacterial treatments, particularly in clinical settings where biofilm-associated infections pose significant treatment challenges. Our study highlights the potential of CAM/TMA-DPH staining as a valuable tool in drug development and biofilm research, providing reliable insights by directly targeting cell viability. This approach is especially timely given the rise of antibiotic-resistant biofilms in chronic infections, such as those linked to cystic fibrosis and endocarditis. Supporting this, recent research (Kolpen et al., 2022), emphasizes the importance of evaluating cellular activity in biofilms, revealing that biofilms in chronic lung infections exhibit lower metabolic rates than those in acute infections, contributing to heightened treatment resistance.

Furthermore, the CAM/TMA-DPH staining method may be particularly suitable for studies exploring alternative antibiofilm compounds. Its stable fluorescence, combined with the elimination of multiple wash steps, allows for real-time monitoring of biofilm dynamics during treatment. This approach can provide valuable insights into the progression of biofilm disruption and bacterial eradication over time.

Despite the advantages of the CAM/TMA-DPH staining approach, some limitations should be acknowledged. Firstly, the potential overestimation of viability in specific bacterial species due to residual esterase activity (Hiraoka and Kimbara, 2002), or the presence of proteases capable of hydrolyzing ester bonds remains a concern (Lee and deMan, 2018). Proteases, which can retain activity after cellular metabolism has ceased, may lead to misleading results. This limitation can be mitigated by conducting parallel CFU/mL analysis to validate viability assessments.

Secondly, the reliance on metabolic activity as a viability marker renders CAM/TMA-DPH staining ineffective for identifying non-metabolic biofilm populations, such as dormant cells. Although TMA-DPH theoretically targets membrane integrity, it cannot differentiate dormant populations from dead cells, emphasizing the need for advanced assays that better discriminate among biofilm subpopulations.

Finally, the structural and phenotypic diversity of biofilms poses a challenge to the universal applicability of this method. While this study demonstrated efficacy across biofilms formed by four clinically relevant species, further research is needed to validate its performance on a broader range of isolates, especially those with atypical biofilm characteristics (e.g., robust but thin or loose but thick structures).

In conclusion, the CAM/TMA-DPH staining method offers a compelling alternative to traditional membrane integrity-based viability assays, particularly when the assessment of biofilm viability requires a focus on metabolic activity. By emphasizing cellular activity, this method delivers valuable insights into the physiological state of biofilm-embedded bacteria, which is essential for both fundamental biofilm research and the clinical management of biofilm-associated infections.

## Data Availability

The raw data supporting the conclusions of this article will be made available by the authors, without undue reservation. For further inquiries, please contact the corresponding author.
