# Supplementary material for: CAM/TMA-DPH as a promising alternative to SYTO9/PI for cell viability assessment in bacterial biofilms
Source: Front Cell Infect Microbiol. 2025 Jan 21;14:1508016. doi: 10.3389/fcimb.2024.1508016 (PMC11790577; doi:10.3389/fcimb.2024.1508016)
Supplement: Supplementary file 1 [file Table1.pdf]

Supplementary information

CAM/TMA-DPH as a promising alternative to SYTO9/PI for cell viability assessment in bacterial biofilms

Tinatini Tchatchiashvili[1,2*], Mateusz Jundzill [1,2], Mike Marquet [1], Kamran A. Mirza [1,2], Mathias W. Pletz[1,2], Oliwia Makarewicz [1,2], Lara Thieme [1,2]

[1]Institute of Infectious Disease and Infection Control, Jena University Hospital, Friedrich-Schiller-University Jena, Jena, 07747, Germany

[2]Leibniz Center for Photonics in Infection Research, 07747 Jena, Germany

*** Correspondence:**

Tinatini Tchatchiashvili

Tinatini.tchatchiashvili@med.uni-jena.de

Supplementary Table 1: Quantitative results obtained from CFU counting and microscopic analysis of biofilm surface area coverage and their correlation as
Spearman rank (*r*). Staining results are presented as mean values ± standard deviation.

Bacterial strain	Treatment	Concentration in mg/L	CFU / mL	Calcein stained biofilm		SYTO9 – PI-stained biofilm	
				Area coverage	Spearman rank (*r*)	Area coverage	Spearman rank (*r*)
P. aeruginosa MIBI685	ciprofloxacin	0	6.05×10^7	56.80% ± 8.33%	0.91 (****)	0.56% ± 0.14%	0.65 (*)
		1.25	7.90×10^5	48.48% ± 3.57%		0.31% ± 0.08%	
		2.5	5.31×10^5	45.25% ± 1.48%		0.20% ± 0.07%	
		10	8.45×10^4	30.98% ± 6.99%		0.24% ± 0.03%	
K. pneumoniae IIMK 217	ciprofloxacin	0	2.35×10^8	25.23% ± 12.35%	0.59 (*)	1.0% ± 0.20%	– 0.04 (ns)
		2	5.59×10^5	2.30% ± 0.77%		2.50% ± 1.70%	
		4	2.48×10^5	1.0% ± 0.3 %		1.71% ±1.28%	
		8	2.32×10^5	1.58% ±1.27%		7.71% ±5.69%	
S. aureus ATCC 43300	vancomycin	0	7.24×10^8	85.85% ± 2.42%	0.88 (****)	70.2% ± 6.99%	0.74 (**)
		512	1.18×10^8	30.42% ± 3.97%		8.31% ± 2.50%	
		2048	6.22×10^7	26.76% ± 7.63%		36.6% ± 5.94%	
		10000	5.50×10^6	0.34% ± 0.45%		3.18% ± 1.45%	
E. faecium MIBI701	gentamicin	0	5.45×10^7	4.13% ± 1.26%	0.83 (****)	5.37% ± 1.59%	0.79 (***)
		64	5.55×10^6	1.38% ± 0.38%		0.75% ± 0.54%	
		256	8.21×10^5	0.62% ± 0.11%		0.57% ± 0.54%	
		1024	4.78×10^4	0.51% ± 0.11%		0.17% ± 0.08%	

r = Spearman correlation rank. Significance was assumed at the *p* value < 0.05 and was indicated by asterisks: * < 0.05, ** < 0.01, *** < 0.001, **** < 0.0001, ns = not significant.
